# The Geographic Distribution of *Saccharomyces cerevisiae* Isolates within three Italian Neighboring Winemaking Regions Reveals Strong Differences in Yeast Abundance, Genetic Diversity and Industrial Strain Dissemination

**DOI:** 10.3389/fmicb.2017.01595

**Published:** 2017-08-24

**Authors:** Alessia Viel, Jean-Luc Legras, Chiara Nadai, Milena Carlot, Angiolella Lombardi, Manna Crespan, Daniele Migliaro, Alessio Giacomini, Viviana Corich

**Affiliations:** ^1^Interdepartmental Centre for Research in Viticulture and Enology, University of Padova Conegliano, Italy; ^2^SPO, INRA, SupAgro, Université de Montpellier Montpellier, France; ^3^Department of Agronomy, Food, Natural Resources, Animals and the Environment, University of Padova Legnaro, Italy; ^4^Consiglio per la Ricerca in Agricoltura e l'analisi dell'Economia Agraria—Centro di Ricerca per la Viticoltura e l'enologia Conegliano, Italy

**Keywords:** *Saccharomyces cerevisiae* native populations, mtDNA RFLP analysis, microsatellite typing, geographic distribution, industrial wine yeasts, winemaking

## Abstract

In recent years the interest for natural fermentations has been re-evaluated in terms of increasing the wine *terroir* and managing more sustainable winemaking practices. Therefore, the level of yeast genetic variability and the abundance of *Saccharomyces cerevisiae* native populations in vineyard are becoming more and more crucial at both ecological and technological level. Among the factors that can influence the strain diversity, the commercial starter release that accidentally occur in the environment around the winery, has to be considered. In this study we led a wide scale investigation of *S. cerevisiae* genetic diversity and population structure in the vineyards of three neighboring winemaking regions of Protected Appellation of Origin, in North-East of Italy. Combining mtDNA RFLP and microsatellite markers analyses we evaluated 634 grape samples collected over 3 years. We could detect major differences in the presence of *S. cerevisiae* yeasts, according to the winemaking region. The population structures revealed specificities of yeast microbiota at vineyard scale, with a relative Appellation of Origin area homogeneity, and transition zones suggesting a geographic differentiation. Surprisingly, we found a widespread industrial yeast dissemination that was very high in the areas where the native yeast abundance was low. Although geographical distance is a key element involved in strain distribution, the high presence of industrial strains in vineyard reduced the differences between populations. This finding indicates that industrial yeast diffusion it is a real emergency and their presence strongly interferes with the natural yeast microbiota.

## Introduction

*Saccharomyces cerevisiae* is the microbial agent responsible for the fermentation of wine, beer and other alcoholic beverages, and the most commonly used microbial leavening agent for bread. Therefore, the evolution of such a yeast is deeply linked to human technological practices. Recently, the impact of human activity on yeast diversity has been assessed at gene and genome level (Fay and Benavides, 2005; Legras et al., 2007; Liti et al., 2009; Schacherer et al., 2009) evidencing several events of domestication (Fay and Benavides, 2005). The presence of *S. cerevisiae* strains is not solely associated to fermentations but also to natural resources, such as vineyard grapes and other fruits (Mortimer and Polsinelli, 1999; Robiglio et al., 2011; Wang et al., 2012; Hyma and Fay, 2013; Knight and Goddard, 2015), insects (Stefanini et al., 2012), oak fluxes or soil associated with oak and other broad-leafed trees (Naumov et al., 1998; Sniegowski et al., 2002; Johnson et al., 2004; Sampaio and Goncalves, 2008; Zhang et al., 2010; Wang et al., 2012; Hyma and Fay, 2013).

Extensive ecological surveys using molecular identification methods have been carried out to explore yeast genetic diversity. Different molecular tools have been used for that purpose and the use of microsatellite offered significant advances in understanding *S. cerevisiae* population dynamics.

Microsatellite analysis has been used to study the genomic diversity of *S. cerevisiae* strains isolated from a natural microsite in Israel (Ezov et al., 2006), to assess the genetic diversity and population structure of native autochthonous *S. cerevisiae* strains, isolated in various Brazilian sugar mills (Antonangelo et al., 2013). Recently microsatellite analysis has been used to study population phylogeny of *S. cerevisiae* strains isolated from human feces (De Filippo et al., 2014), to analyze *S. cerevisiae* strains isolated from palm wine samples in Burkina Faso and compare them with other strains with different origins (Tapsoba et al., 2015) or to characterize *S. cerevisiae* strains isolated from the oak niche in different regions of the Mediterranean and Japan and to compare them to 144 reference genotypes (Almeida et al., 2015). Microsatellite analysis has been used to differentiate the population genetic structure of wine and vineyard *S. cerevisiae* strains (Legras et al., 2007; Schuller and Casal, 2007). Recently Franco-Duarte et al. (2014) used polymorphic microsatellites to genetically characterize a wide group of *S. cerevisiae* strains, from different geographical origins and technological groups, and related their data with enologically important phenotypic traits.

Mitochondrial DNA (mtDNA) represents only a small fraction of yeast genome size, yet it has been by far the most popular marker of yeast molecular diversity. Particularly the RFLP mtDNA has been used for yeast strain genotyping as mitochondrial DNA is highly variable in natural populations (Querol et al., 1992). Its elevated mutation rate can generate some signal about population history over short time frames. It is widely considered that mtDNA is maternally transmitted, and therefore clonal, in animals. On the contrary in yeast the prevalent generation of recombinant mitochondrial genomes occures, but rarely mtDNA can be inherited uniparentally (Berger and Yaffe, 2000). Both methods were successfully applied to oenological environment. In particular they have been used for the characterization of vineyard-associated *S. cerevisiae* strain diversity revealed by fermentation of grape samples or direct isolation from grapes (Valero et al., 2007; Capece et al., 2012; Schuller et al., 2012). MtDNA has been widely applied for yeast identification at strain level of *S. cerevisiae* from wine and other enological environments (Comi et al., 2000; Santamaría and Bayman, 2005; Di Maio et al., 2012; Bovo et al., 2016). Although extensive ecological surveys are already present in literature, unraveling the forces shaping *S. cerevisiae* population structure and genetic diversity remains one of the main challenges of the research on yeast ecology. In fact the dynamics of strain differentiation are still partially known and the exploration of new ecological niches can reveal alternative sources of yeasts with innovative features to be used for biotechnological purposes.

Recently, study on genetic diversity of vineyard populations and strain identification shed a light on the impact of the use of industrial wine yeasts on vineyard native microbiota. Few strains, mainly selected in wineries during natural fermentation and commercialized, have been widely used for winemaking for last decades (Valero et al., 2007). These starters are used without any special containment and can be annually released in large quantities, together with liquid and solid winemaking residues, in the environment around the winery. A first study led in two different wine producing areas in France and Portugal, revealed a limited dissemination of commercial yeasts in the vineyard, restricted to short distances and periods of time (Valero et al., 2005). Another study limited starter strain survival in vineyard to 3 years on the vine (Cordero-Bueso et al., 2011). However, the lower yeast diversity observed in Chile in areas using industrial yeast starters in comparison to autochthonous fermentation suggests that industrial yeasts disseminate outside the cellar (where they are used; Cubillos et al., 2009). Therefore, preserving biodiversity is important in order to ensure the conservation of gene pools of technological importance and this point should be further investigated.

In this study we led a wide scale investigation of *S. cerevisiae* diversity and population structure in three neighboring winemaking regions of Protected Appellation of Origin, in North-East of Italy. In each region the most represented grape variety was considered, and all samples were collected during the same harvest. From 634 grape samples collected over 3 years, we could detect strong variability in the presence of *S. cerevisiae* yeast, according to the winemaking region. With the aim of evidencing the presence of geographic differentiations among strains in very close winemaking area and the extent of wine yeast dissemination in vineyard, two methods have been used. MtDNA RFLP analysis was performed to differentiate yeast isolates. The allelic variation at 18 microsatellite loci has been studied to assess the genetic diversity and population structure.

## Materials and methods

### Wine making areas, grapevine varieties, and sampling

**Figure 1:**
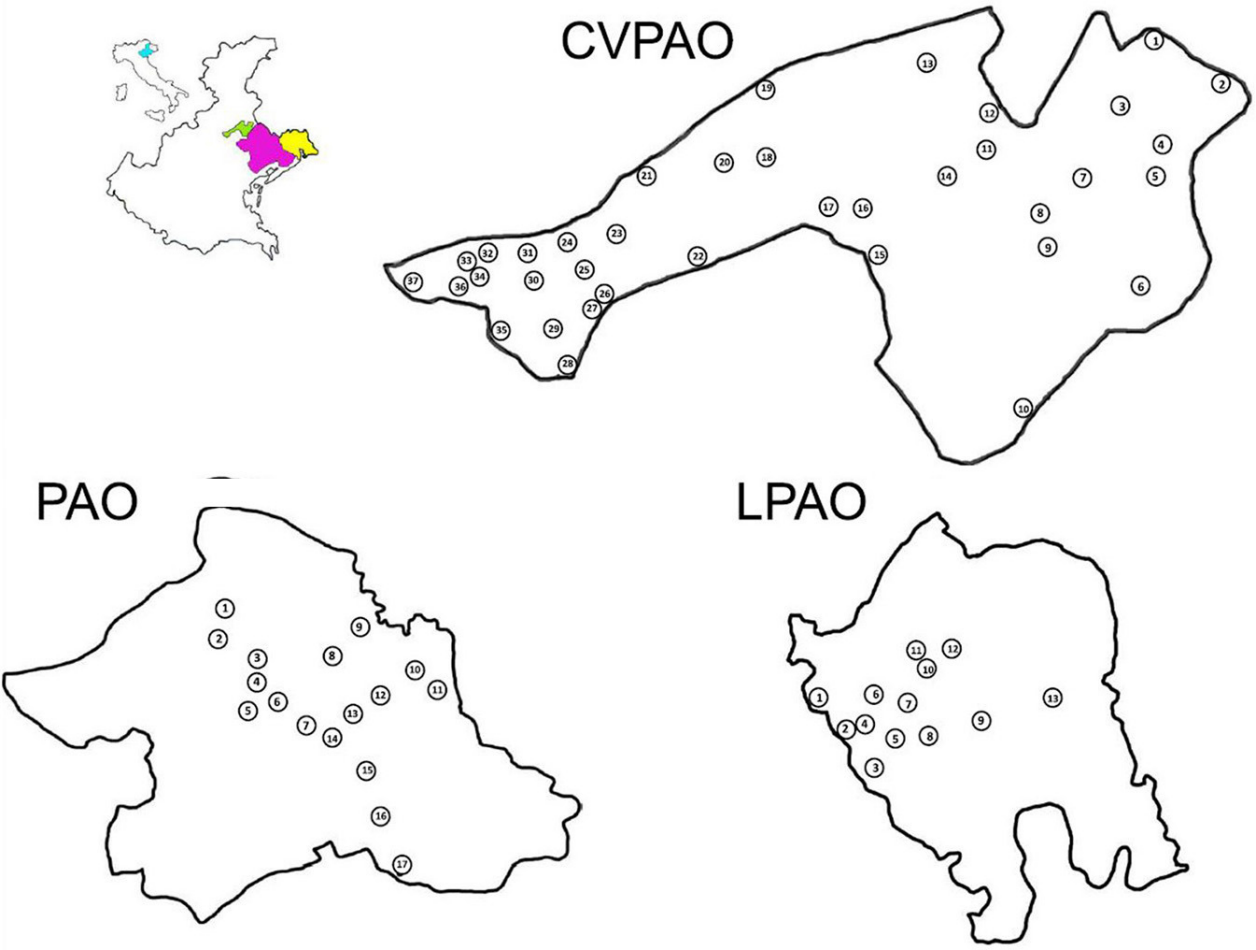
Geographic distribution of the sampled subareas in CVPAO, LPAO, and PAO. In each of the Protected Appellation of Origin regions the subareas are numbered: CVPAO from 1 to 37 (in the text Pr01-Pr37), LPAO from 1 to 17 (in the text To01-To17) and PAO from 1 to 13 (in the text Ra01-Ra13). Each region was divided into subareas to obtain a homogeneous sampling.

Grape bunch samples of local grape varieties were collected during three sampling campaigns in the vineyards of three neighboring winemaking regions in the North-East of Italy: Conegliano–Valdobbiadene Prosecco superior (CVPAO), sampled in 2004, Piave (PAO) sampled in 2006 and Lison-Pramaggiore (LPAO), sampled in 2010. For each of the three winemaking regions the most relevant grape variety was taken into account: Glera in CVPAO, Tocai friulano in LPAO and Raboso Piave in PAO. The first two are white grape varieties with great vegetative force and good production (average pH of 3.2 in Glera and 3.3 in Tocai friulano, that reach 10.5 and 12% alcohol degrees, respectively) while Raboso Piave is a red variety with strong acidity and astringency (average pH of 3.0 and 12% alcohol degrees). Samplings were performed in the vineyards of 162 different wineries as reported in Table 1. Grape bunch samples were collected from 3 to 5 days before harvest and samplings were organized in order to cover the whole areas where the selected grape varieties were cultivated. Each area was divided into subareas to obtain a homogeneous sampling (Table 1). The different number of sampled subareas was due to the diffusion in each region of the selected varieties. For each subarea generally 2 vineyards were sampled collecting an average of 4 bunches (fermented separately) per each vineyards. The geographic location of the sampled subareas is reported in Figure 1.

**Table 1:** Features of the winemaking area and sampling.

	**Size of the Appellation of Origin area (km^2^)**	**Wineries**	**Subareas**	**Grape bunches for each area**
CVPAO	200	97	37	353
LPAO	700	45	13	203
PAO	1,350	20	17	78
Total	–	162	67	634

### Fermentation, strains isolation, and identification

The collection has been made, at each stage, avoiding touching the grapes with hands and sterilizing scissors periodically in order to minimize contamination. Samplings were carried out choosing only healthy bunches (with no symptom of mold infection). Stomacher sterile bags were used, filled with about 700–800 g of grapes (corresponding to one or two bunches, depending on size). Samples collected in the vineyard were transferred to the laboratory where half fructose and half glucose corresponding to 2% of grapes weight were added with 500 μl of sulfur dioxide at 5% v/v (both to stimulate the development of *Saccharomyces* strains). Each sample was then manually pressed and left to ferment spontaneously at room temperature for 2–3 weeks with skins and stalks. The fermentation process was considered completed when the weight loss, monitored measuring bags weight daily, was stable. After fermentation, diluted samples (from 10–4 to 10–6) were spread on WL plates (Oxoid) and, after an incubation of 2 days at 25°C, 16 colonies with *Saccharomyces*-like morphology (cream to light green color, round shape, umbonate and smooth colony on WL medium) were randomly collected. The isolates were stored at −80°C in glycerol (40% v/v).

Multiplex PCR (Nardi et al., 2006) and ITS restriction analysis (Esteve-Zarzoso et al., 1999) have been used to identify *S. cerevisiae*. DNA extraction and PCR conditions were carried out as reported by Bovo et al. (2009).

### mtDNA RFLP analysis

The method developed by Querol and Ramon (1996) was chosen. Yeasts colonies obtained on YM agar medium, after incubation for 48 h at 25°C, were resuspended in 1 ml of sterile water and then centrifuged at 14,000 rpm for 3 min in an Eppendorf microcentrifuge. DNA extraction, *Hin*fI digestion and electrophoretic runs were carried out as reported by Bovo et al. (2011).

### Microsatellites analysis

#### Strains and DNA isolation

All the strains with different mtDNA profile were analyzed by means of 18 microsatellite loci. When the same profile was found in more than one sample, one strain from each sample was taken into account.

In order to better understand yeasts evolution, the survey was also conducted on 37 commercial strains, coming from different substrates like wine, sake, ragi, beer, oak, bread, laboratory, and clinical (see Supplementary Material, Table S1).

Yeast cells were cultivated in 5 ml YPD medium (36 h at 25°C, 150 rpm) and genomic DNA was extracted using E.Z.N.AR yeast DNA kit (OMEGA Bio-Tech, USA).

#### Microsatellites amplification

To achieve this analysis, 18 microsatellite loci (Perez et al., 2001; Legras et al., 2005; Richards et al., 2009) were combined in two sets of nine loci (see Supplementary Material, Table S4) labeled with different fluorogenic dyes and amplified using the Type-it Microsatellite PCR kit (QIAGEN, Milan, Italy). PCR reactions were run in a final volume of 12.5 μl containing 10 ng of yeast DNA. Amplification was performed using a Gene Amp 9700 (Applied Biosystems, Monza, Italy) thermal cycler under a three-stage temperature program: stage one, 95°C – 15 min, stage two (34 cycles) 95°C – 30 s, 57°C – 2 min, 72°C – 1 min, stage three: 60°C – 30 min.

#### PCR product analysis

PCR products were sized for 18 microsatellite loci on a capillary DNA sequencer (ABI 3130 XL, Applied Biosystems) with the DS-33 Matrix Standard Kit (Dye Set G5, Applied Biosystems) using the polyacrylamide Pop7 and the size standard GeneScan 500LIZR (Applied Biosystems). Before the analysis, the PCR amplicons were first diluted 100-fold and then 0.5 μl of the dilution was added to 9.35 μl of formamide (Applied Biosystems) and 0.15 μl of GeneScan 500LIZR size marker, and the mixture was denaturated at 95°C for 5 min. Raw size were assigned into classes of alleles of similar size (±1 bp for trinucleotidic repeats and 0.75 bp for dinucleotidic repeats) using GeneMapper software version 4.1 (Applied Biosystems). Data are given in Table S5.

### Population analysis

In order to assess the relationships between strains, we have used the Bruvo's genetic distance (Bruvo et al., 2004) which is similar to band-sharing indices used with dominant data but takes into account mutational distances between alleles. This distance requires perfect microsatellite loci, which is almost the case for all the loci except C4, which is composed of two motifs. Nevertheless, locus C4 represent only one locus over 18. Moreover, the use of Bruvo distance with 12 microsatellite loci (Legras et al., 2007) clearly improved the phylogenetic signal of the initial dendrograms obtained with DC chord distance (Legras et al., 2017), making it closer to phylogenies obtained with whole genome data. Therefore, we have chosen this genetic distance, despite the potential violation of the initial model proposed by Bruvo et al. (2004).

The Bruvo distance (Kamvar et al., 2013) was calculated between each strain with the POPPR (v1.1.5) package under R statistical software v3.13 (R Core Team, 2012). Trees were obtained from distance matrices with ape v3.3 R package, drawn using Mega 5.05 (Kumar et al., 2004) and rooted by the midpoint method. The reliability of the tree topologies was assayed through a jackknife procedure and the consensus nodes given by Mega 5.05. In addition a network was drawn using SPLITSTREE4 V4.10 (Huson and Bryant, 2006). Ancestry was inferred using InStruct (Gao et al., 2007) on the set of wine and strains from other origins. The results of 20 run for values from *K* = 5 to 15 have been compared and the best DIC value was chose. The results of these 20 runs were combined with CLUMP according to the LargeKGreedy method.

Population analyses were performed with Fstat version 2.9.3 software (http://www.unil.ch/izea/softwares/fstat.html) for Weir and Clark Cockerham (1984) estimates of Fst. Population differentiation was tested according to OBSTRUCT. In order to avoid the impact of the presence of strains related to industrial starters, all strains presenting a Bruvo distance <0.2 with one of the yeast starters used in these area were removed from the dataset. This left 172 isolates, originating from 22 areas with at least 3 strains. We combined single or pairs of strains of 7 sites with those of the nearest site. These 172 wine strains were again analyzed with InStruct and an optimum K of 10 populations was obtained. The results of 9 runs were combined with CLUMP for OBSTRUCT analysis.

Outcrossing rates were estimated from the heterozygosity profile (Johnson et al., 2004) with a mathematica script kindly provided by. M. Goddard, and as 1- the selfing rates estimated from Fis values (Wright, 1969). Fis was estimated with Fstat.

## Results

### *Saccharomyces cerevisiae* strain collection obtained from spontaneous fermentation of grape bunches collected in vineyards

Grape bunch samples of local grape varieties were collected in the vineyards of three neighboring winemaking regions in the North-East of Italy: in Conegliano–Valdobbiadene Prosecco superior (CVPAO) vines are mainly grown on hillside, although at low altitude varying between 50 and 500 m above sea level, in Lison-Pramaggiore (LPAO) and Piave (PAO) vines are cultivated on plain areas. All the three regions, started as traditionally artisanal winemaking regions, nowadays adopted industrial practices, such as extensive use of fungicides and commercial yeast strains for fermentation. In CVPAO Glera variety has been sampled, a white variety that generally reaches a sugar content of about 160 g/l, in LPAO Tocai friulano, a early-ripening white variety that reaches 230 g/l, and in PAO Raboso Piave, a late-ripening red variety (the grape is generally harvested 1.5–2 months later than the others) with an average sugar content of 220 g/l. A total of 634 bunches were collected, 4 per each vineyard. From each single bunch fermentation the yeast isolates were identified at species level in order to determine *S. cerevisiae* presence in vineyard (Table 2).

**Table 2:** Presence of *S. cerevisiae* in the fermented grape bunches collected from the three winemaking regions.

	**Grape bunches**	**Grape bunches containing *S. cerevisiae***	**Percentage of grape bunches with *S. cerevisiae***
CVPAO	353	30	8.5
LPAO	203	18	8.6
PAO	78	54	69.2

Despite the huge number of grape bunches collected from the two white varieties, 203 in LPAO and 353 CVPAO, the presence of *S. cerevisiae* in vineyards is very limited. On the contrary *S. cerevisiae* seems to be much more abundant in the vineyards of the red variety (PAO).

**Figure 2:**
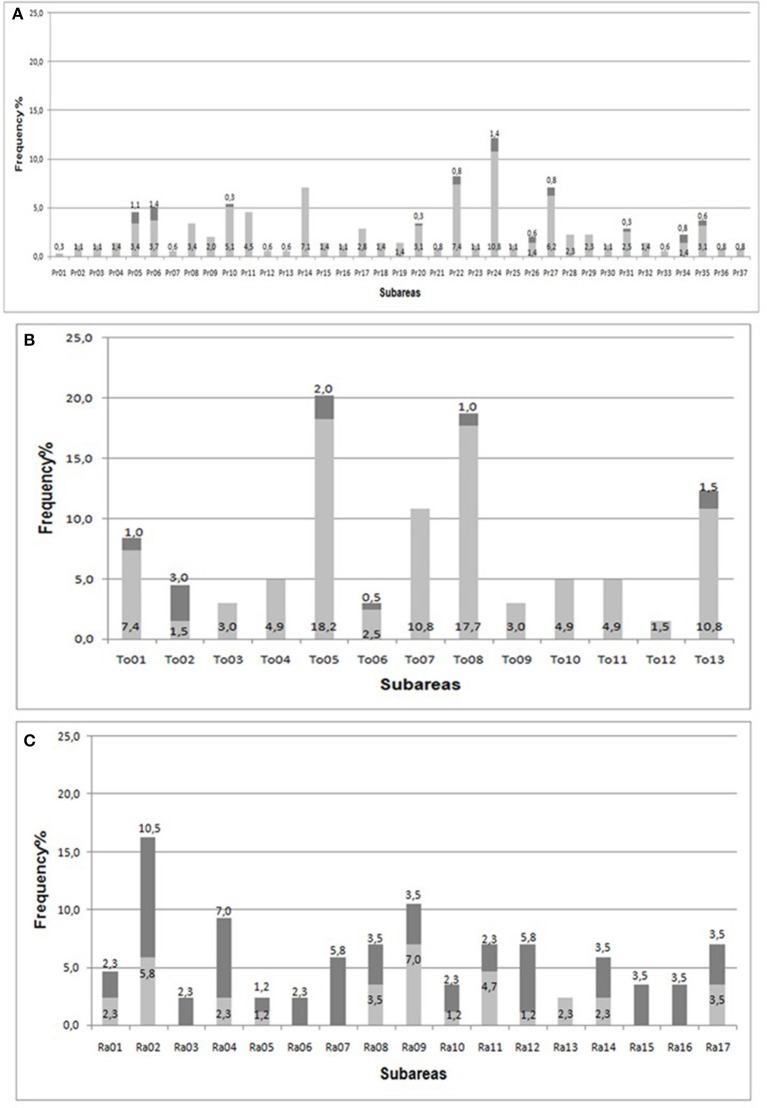
Frequency of the fermented grape bunches containing yeasts belonging to the genus *Saccharomyces* (dark gray) and without (light gray), collected in each subareas of CVPAO **(A)**, LPAO **(B)**, and PAO **(C)**.

The occurrence of *S. cerevisiae* in CVPAO sampled subareas is very irregular: only 11 sites out of 37 allowed the isolation of *S. cerevisiae* (Figure 2A). Similar results were obtained for LPAO where in more than 50% of sampled sites, seven out of thirteen, no *S. cerevisiae* strains were recovered (Figure 2B). Most of the vineyards carrying *S. cerevisiae* are located in the western part of the Appellation of Origin area. In this case the distribution of the vineyards containing *S. cerevisiae* strains is less homogeneous.

In contrast with the two former vineyards, most of grape bunches sampled in PAO area contained yeasts belonging to *S. cerevisiae* species, with 11 positive sites out of 17 (Figure 2C).

### Evaluation of yeast diversity by mtDNA RFLP analysis

By means of Multiplex PCR and ITS restriction analysis 295, 197, and 254 isolates from CVPAO, LPAO, and PAO, respectively, have been identified as belonging to *S. cerevisiae* species. In CVPAO 1.9% of the isolates were identified as *S. paradoxus*.

In order to discriminate the different yeast genotypes, mtDNA RFLP analysis has been performed (Querol and Ramon, 1996; Lopez et al., 2001; see Figure S1 for mtDNA RFLP dendrogram). A total of 183 different mtDNA genotypes has been found. The mtDNA RFLP analysis evidenced the presence of 17 different profiles from 197 isolates for LPAO and 37 from 295 isolates for CVPAO while, they were 129 from 254 in PAO (Table 3), thus evidencing the low level of genetic diversity, in terms of genotype number, of LPAO and CVPAO in comparison to PAO. Kruskal-Wallis one-way analysis of variance confirmed that in PAO the number of genotypes per sampled sites was statistically different (*P* < 0.05) compared to CVPAO and LPAO.

**Table 3:** Number of different profiles obtained by mtDNA RFLP analysis for each sampled area.

	**Number of colonies**	**mtDNA RFLP profiles**	**Profiles/ *S. cerevisiae* ratio**
CVPAO	295	37	1:7
LPAO	197	17	1:11
PAO	254	129	1:2

The mtDNA RFLP profiles obtained for 69 commercial strains that are commonly used in the wineries of these winemaking regions were added to the dataset, and cluster analysis was also performed. Profile comparison showed in some cases 100% similarity to vineyard isolates, as described in Table 4.

**Table 4:** Presence of industrial strains (IND) in the three winemaking regions.

**Profile**	**IND**	**Subarea**	**Percentage of IND in the subarea**	**Percentage of IND on total *S. cerevisiae* isolates**
T7	Intec Ever Mycoferm 611	To01	33	2
P6	Lalvin D47	To01	22	13.3
	FR95	Pr27	41	
		Pr05	83	
		Pr34	38	
		Pr06	43	
		Ra06	11	
P11	Vason Premium Blanc12V	Pr24	18	7
		Pr27	20	
		Pr35	82	
		Pr05	17	
P13	LV10	Pr06	29	0.4
P36	Vason Nouveaux Ferments	Ra06	11	4.5
	EC1118	Pr24	27	
	QA23	Ra14	54	
	DV10	Ra02	7	
		Ra03	11	
		Ra12	18	
		Ra10	40	
		Ra09	24	

Interestingly, the profile P6, corresponding to the commercial strains FR95 and D47, was found in all three Appellation of Origin areas indicating a high diffusion of this profile among the vineyards, while P11 (Vason Premium Blanc 12V), P13 (LV10) and P36 (Vason Nouveaux Ferment, QA23, DV10, and EC1118) were diffused in CVPAO and PAO. All these commercial strains are frequently used in the local wineries. The frequency of profile P36 is the most striking as it has been isolated in 7 different subareas of PAO, and in one of CVPAO. The percentage of isolates that shared the mtDNA profile with commercial strains reached 27.5% of the total number of stains that were characterized. In particular more than half of colonies isolated in CVPAO (51.5%) had the commercial strains profiles while the percentage was 22.6% in LPAO and only 9.5% in PAO.

**Figure 3:**
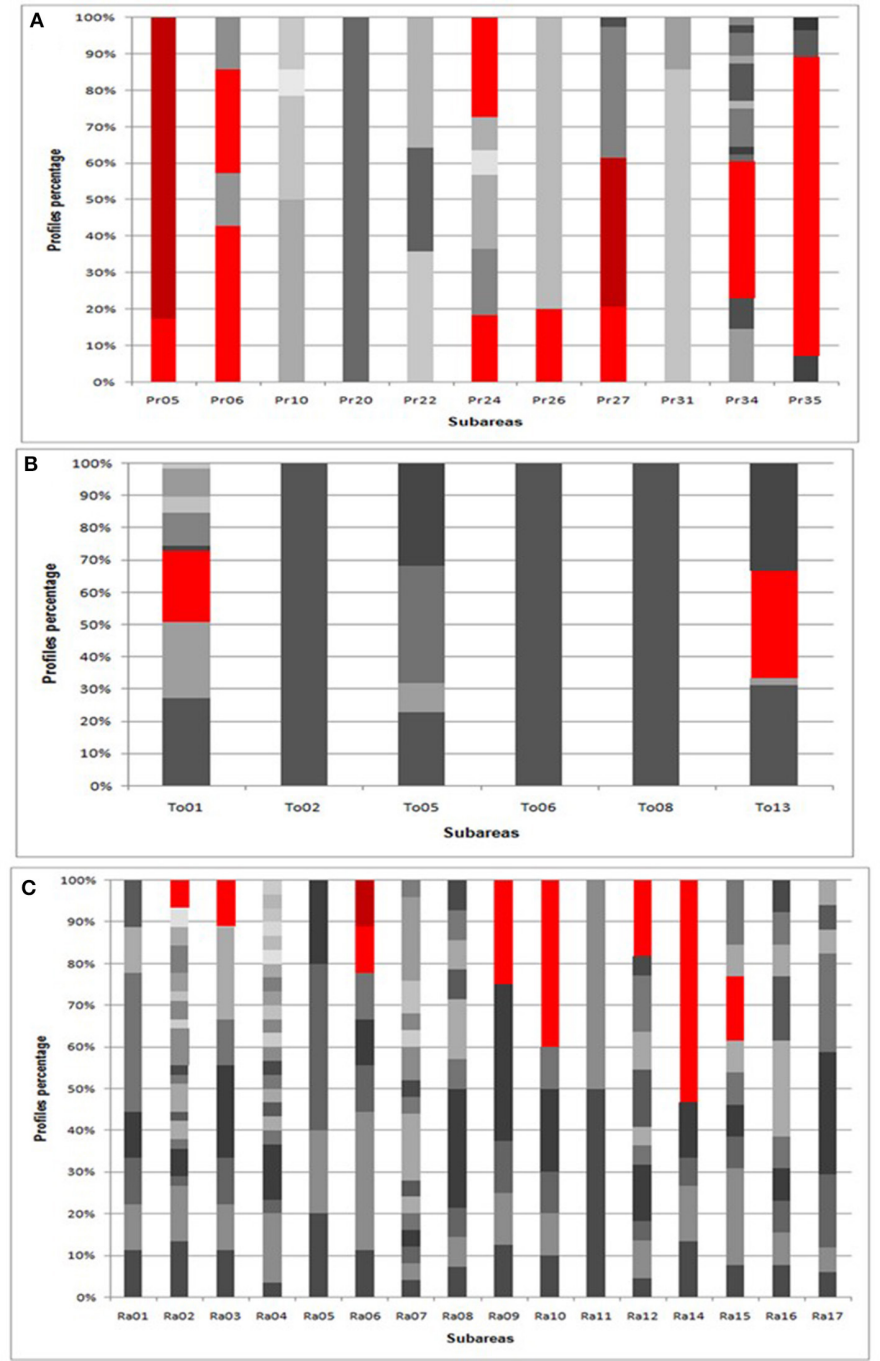
Percentage of mtDNA RFLP profiles for each subareas in CVPAO **(A)**, LPAO **(B)**, and PAO **(C)**. In each subarea the percentages of different profiles are indicated by different shades of gray. The commercial profiles percentages are reported in red.

The comparison of mtDNA profiles of isolates from each subareas (Figure 3), revealed that genotypes different from the commercial profiles are specific or possibly shared by few sampling sites. When a shared genotype occurred, it was found preferentially in not neighboring subareas. In particular in CVPAO all of the autochthonous profiles were encountered once. In LPAO only profile T2 was found in more than one subarea (three). Among them two were neighboring. In PAO 8 profiles were found in more than one subarea. Regarding R6, R7, R112 each profile was isolated in three different subareas. As regard R6 and R7 all the subareas were not neighboring with other sampling sites in between. On the contrary in the case of R112 two of the three subareas were neighboring. In the other cases (R15, R21, R27, R50, and R70 profiles) each profile was found in two specific subareas. Only in the case of R15 and R50 the subareas were neighboring. In some of the subareas present in CVPAO and PAO the profiles of the commercial strains (in red in the graph) were found at higher number than the autochthonous ones.

Moreover in LPAO only half of the subareas carried more than one profile. In CVPAO only one single profile subarea was found (Pr20) while in the others two to eleven profiles were detected. In PAO only one subarea showed two profiles while in the others the number of different profiles ranged from 3 to 23.

### Evaluation of yeast diversity from microsatellite polymorphisms

#### Strains characterization with microsatellite markers

In order to understand the genetic relationships among the strains isolated in the three different wine producing areas, a total of 219 autochthonous isolates was analyzed at 18 microsatellites loci. This includes the 183 different genotypes obtained by mtDNA RFLP analysis. When the same genotype was found in more than one fermented grape bunch, one yeast isolate from each bunch was taken into account. Since we have isolated strains from bunch of grapes using an enrichment procedure and due to the scarcity of yeast on sound grapes (Martini et al., 1996; Mortimer and Polsinelli, 1999) we made the hypothesis that isolates with identical mtDNA profiles were likely to originate from the same cell (clonal replication). This suggests that the evaluation of yeast diversity obtained with microsatellite analysis might be slightly under estimated. Isolates sharing the same mtDNA profile of commercial strains were also considered. Simultaneously, 34 strains were genotyped with the same set of markers. These strains include 8 commercial wine strains and 26 strains whose genome has been sequenced. Among them there were 8 commercial wine strains, 2 beer strains (NCYC361 and Clib382), 2 clinical isolates (YJM428 and YJM653), 1 bread (6662), 1 rum (JAY270), 1 laboratory (S288c), 1 oak (NC02), 1 sake (UC5), and 1 ragi (Y9) strains and 8 wine or vine isolates.

**Figure 4:**
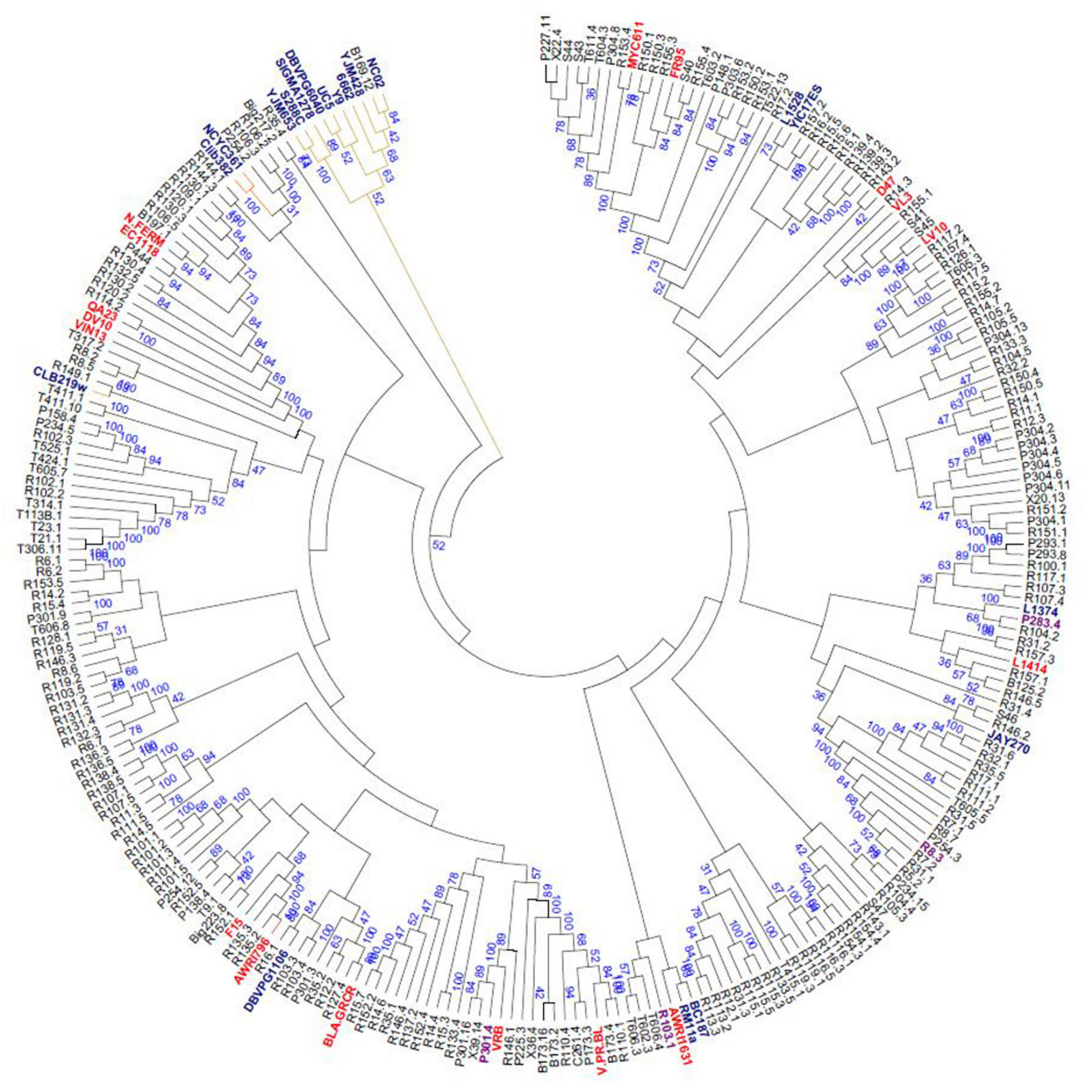
Consensus neighbor-joining tree showing the clustering of 258 yeast strains isolated from different sources. The tree was constructed from the chord distance between strains based on the polymorphism at 18 loci and is rooted according to the midpoint method. All the strains coming from our isolation programs are in black, while commercial strains (in bold font) are in red for wine strains, and in light blue all the rest. The reliability of the nodes is given by the frequencies they were encountered in a Jackknife procedure removing out from the analysis one locus out of 18 locus after the other.

The microsatellites typing has revealed 242 different genotypes out of 258 yeasts analyzed (206 for the vineyard isolates out of 219), whereas 183 different profiles were recovered with the mtDNA RFLP analysis, and only six strains were not differentiated in the survey. Only one of them has shown identical alleles at 18 loci with a commercial wine strain. The 18 microsatellite loci recorded from 7 to 33 different alleles per locus. SCAAT1, SCYOR267c, C5, and C4 displayed the highest number of alleles in the global population, which was expected, given the length of these repeated motifs and their selection for high polymorphism (Legras et al., 2005). The loci C9 and YKL172w showed the lowest polymorphism with 11 and 7 alleles, respectively. The number of alleles per locus per strain varied from one to two for most yeasts analyzed except for 6 which presented a third additional allele at locus C4 and one strain at locus C8. In total 32% of the isolates were homozygous for all loci. A consensus neighbor-joining tree calculated from the Bruvo distance matrix for all pairs of strains and a network are reported in Figure 4 and Figure S2. As the global tree structure is different from mtDNA RFLP profile, we compared the dissimilarity distance matrix obtained on mtDNA RFLP and microsatellite based data by a Mantel test. The Pearson coefficient was *r* = 0.11 indicating that distance matrixes were indeed dissimilar, so that little of the global genetic variation described in each distance matrix is shared. This can be seen when the dendrograms obtained with the two methods are plotted simultaneously (Figure S3).

The tree shows a cluster of strains coming from substrates different from wine: ragi, sake, laboratory (S288C and Sigma1278), clinical isolates and a strain isolated from fermenting fruit juice (DBVPG6040) as described previously (Legras et al., 2007; Erny et al., 2012). These strains have already been described as genetically different from European and wine strains (Liti et al., 2009). This cluster contains the vineyard strain B169.12 isolated from the CVPAO area, closely associated to oak strain NC02.

A second cluster contains 9 strains and is associated to this first cluster. It contains 5 strains isolated from PAO area, 2 from the CVPAO area that has a different genetic background and two strains isolated from beer, namely NCYC361 isolated in Ireland and Clib382 isolated in Japan. This cluster can be considered as a particular one as it contains strains which are poorly related to other wine strains, despite they have been isolated from grape skin.

A third cluster contains 17 strains including 10 strains from the LPAO area, 2 from the CVPAO area, 4 from the PAO area and CLIB219 isolated from *Vitis amurensis* and very different from wine strains (Schacherer et al., 2009).

Commercial wine strains and the control vine isolates are distributed in several clusters of the tree. One of these cluster is made of up to four commercial wine strains, EC1118, DV10, QA23, VIN13, and NOUVEAUX FERMENTS described previously as a group of “Champagne strains” (Legras et al., 2007; Coi et al., 2017) to which are also related 11 yeast strains isolated in PAO area from Raboso Piave grape variety.

Regarding those vineyard isolates sharing the same mtDNA profile as commercial strains, one for each single grape bunch fermentation was taken into account. Among them only one has shown identical alleles at 18 loci with a commercial yeast. Two commercial mtDNA profile are the most widespread, P6 and P36 that correspond to commercial strains FR95/D47 and EC1118/QA23/DV10/Vason Nouveaux Ferments, respectively. In P6 group 12 isolates (T611.4, T603.2, T604.3, X39.14, P303.6, X20.13, X22.4, S44, P227.11, P304.4, R155.3, and S40) have been analyzed. Most of the isolates clustered nearly FR95 (Figure 4), except X20.13 and X39.14. In P36 group 8 isolates (B197.1, R110.4, R132.5, R106.5, R114.2, R120.1, R130.3, and R109.1) have been analyzed. Most of the isolates clustered in the group of the wine commercial yeasts EC1118, QA23, DV10, Vason Nouveaux Ferments, except R110.4.

#### Population structure

*Saccharomyces cerevisiae* life cycle combining clonal expansion and infrequent mating (~1 mating event over 1,000 mitose (Ruderfer et al., 2006; Magwene et al., 2011) which is very likely varying according to environmental conditions.

*Saccharomyces cerevisiae* life cycle combining clonal expansion and infrequent mating (1 mating event over 1,000 mitose, Ruderfer et al., 2006) with high selfing rate, promotes a strong population structure (Wang et al., 2012). In order to infer population structure, we used InStruct software, which takes into account for inbreeding the main the main sexual reproductive mode in *S. cerevisiae*. Deviance information criterion indicated the most likely population number to be *K* = 12 and the results from 20 runs obtained for *K* = 12 were combined with CLUMPP.

**Figure 5:**
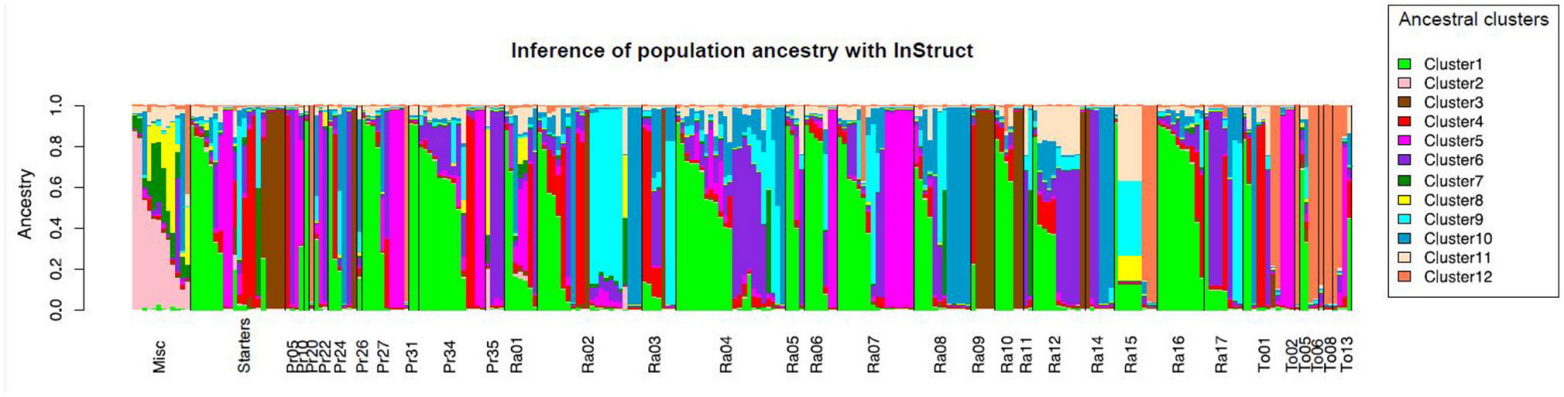
Barplot presenting the population ancestry inferred with InStruct. The results of 20 runs were combined with CLUMPP.

Some individuals could be assigned to 9 ancestral clusters, while the 3 remaining ancestral clusters were partially associated to strains in the sample analyzed here (Figure 5). Some populations were clearly differentiated from others with individuals fully assigned to one ancestral cluster for 6 of the 12 inferred clusters whereas others appeared as admixed. Ancestral cluster 3 was associated to industrial starters EC1118, QA23, DV10, and 7 strains from the PAO area (i.e., R106.5) and ancestral cluster 5 to the industrial strains MYC611 and FR95, and 18 strains from the three areas. Ancestral cluster 1 is associated to the highest number of strain: 42 strains from PAO and CVPAO areas, and one strains from LPAO, as well as to commercial isolates L1414, VL3, LV10, and wine isolates L1374, and DBVPG1106. The sixth ancestral cluster is associated to 15 strains from PAO and CVPAO areas. Interestingly, the last ancestral which also presents the highest selfing rates gathers 15 strains from LPAO area (i.e., T113b1, T314.1…) or PAO area (R102.1-3) and strain CLIB219. Most of the strains not of wine origin were inferred to be associated with ancestral groups 2, 7, and 8: lab strain S288C, and its derivative sigma 1278, sake strains UC5 and Y9, two clinical isolates YJM653 and 428; however two Italian grapes isolates B169.12, and R8.7 were inferred to be associated with these groups.

#### Spatial, geographical, and genetic structure of vine isolates inferred from microsatellite data

As all vineyard isolates have been collected over three different geographic areas is thus logical to question how the location of these populations may have influenced yeast genetic diversity. A first look at Figure 5 indicates that some clusters (related to CLIB219) are almost only found among LPAO areas samples. However, we needed to assess the robustness of these wine strains clusters.

Given the small number of microsatellite genotypes identified in several subareas, Nei's pairwise Fst estimates might have a high variance. A dendrogram built from the Fst distances matrix offers a first overview of genetic differentiation between groups (Figure S4) and reveals that most populations of the LPAO area are separated from PAO (*p* < 0.05, Table S2). For CVPAO and PAO no differentiation could be evidenced between sampling sites (data given as Supplementary Information).

**Figure 6:**
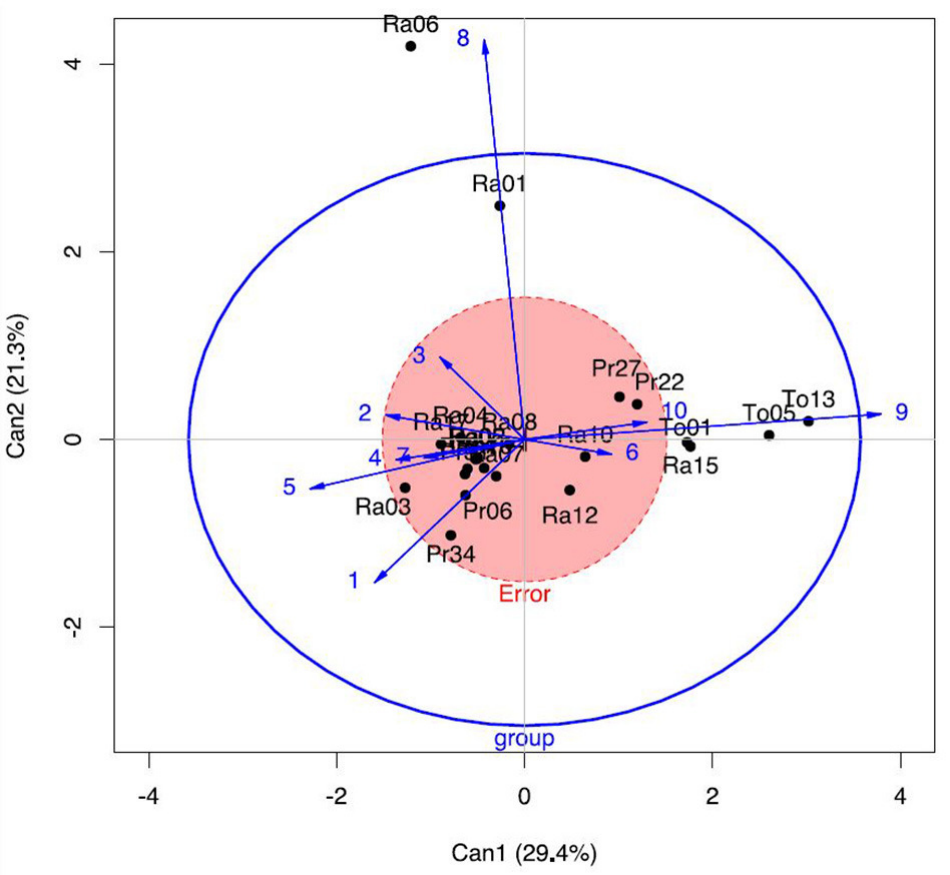
Canonical discriminant analysis of the ancestry obtained for wine isolates of different vineyards with OBSTRUCT, after removal of strains related to industrial starters. The outer blue ellipsoid labeled group reflects the variation of the group means around the grand mean while the red circle reflects the pooled within-group dispersion and covariation (Gayevskiy et al., 2014).

In order to confirm this differentiation of LPAO area from PAO and CVPAO, we used the ancestry based approach as implemented in the OBSTRUCT package (Gayevskiy et al., 2014). The potential effect associated to the release of industrial yeast in the environment, was erased from the removal of strains closely related to industrial starters frequently used in this area, but we also combined strains from close sites given of the reduced number of isolates. OBSTRUCT inferred that several populations could be differentiated from others (Table S3) as for Ra04, Ra12, or P34 areas. In addition a canonical discriminant analysis (CDA) also performed by OBSTRUCT on the ancestry of the different populations (Figure 6) indicates the differentiation of several LPAO sites (To01, To03, and To05) according to the first axis and for two populations of the PAO area (Ra01 and Ra06) according to the second axis. For LPAO, this differentiation correspond to the presence of strains of the twelfth cluster (orange) among populations of To02, To05, To06, To08, To13 sampling sites. These strains are also detected in Ra15 in the PAO area, close to the LPAO area. As a conclusion, CDA and Fst distance matrix analysis suggest a geographic cline in strain diversity between LPAO and PAO.

#### Heterozygosity of the different yeast populations

The number of heterozygote loci detected in the genome of each strain depends on the population life cycle. In order to evaluate if the different group inferred by InStruct have the same life cycle, we estimated the outcrossing rates of the different inferred groups from Fis, or from the heterozygosity profile (Johnson et al., 2004; Table 5).

**Table 5:** Estimates of outcrossing rates from Fis values (Wright, 1969), or and from heterozygosity profile (Johnson et al., 2004) likelihood estimates.

**Outcrossing estimates (%)**	**Cluster1**	**Cluster3**	**Cluster4**	**Cluster5**	**Cluster6**	**Cluster9**	**Cluster10**	**Cluster12**
from Fis	26	100	3.4	55.3	35.1	26.7	2.4	6.4
Johnson et al., 2004	18.9% [13, 27]	82% [75, 88]	4.4% [0.7, 14]	69.2% [43, 100]	34% [17, 57]	14.8% [6.3, 29]	2.5% [0.4, 8.1]	4.6% [1.6, 10.1]

The estimates of outcrossing rates are ranked in a similar manner for both methods. They show quite clearly that strains of cluster 12, related to isolate CLIB219, and found mainly in LPAO populations present a lower heterozygosity corresponding to a higher selfing rate. In contrast to cluster 12, cluster 3 containing yeast starter EC1118 present an outcrossing rate close to 1, in line with our recent finding that EC1118 group results from a cross between a typical wine and a flor isolate (Coi et al., 2017).

## Discussion

Inside vineyard microbiota, *S. cerevisiae* populations have a rather small size, especially on healthy berries (Mortimer and Polsinelli, 1999). As *S. cerevisiae* is the main agent of alcoholic fermentation, many studies have been focused on that yeast, and *S. cerevisiae* abundance and genetic differences in grape must have been correlated with agricultural practices (Cordero-Bueso et al., 2011), grape variety, geographical distances (Schuller et al., 2012) and vineyard diffusion of commercial starters (Valero et al., 2005, 2007). However, the differentiation of yeast populations isolated from diverse regions has been suggested recently (Legras et al., 2007), and, a later time, proven (Gayevskiy and Goddard, 2012).

To better investigate how geographical distances influence yeast genetic variability, in this study *S. cerevisiae* population diversity of three contiguous Appellation of Origin areas have been analyzed. In order to obtain the highest number of yeast genotypes present in the vineyards we performed a widespread sampling of grape bunches and single grape bunch fermentation were carried out, before yeast isolation. Fermentation conditions were need for *S. cerevisiae* isolation as this yeast is poorly present on the grape surface. Firstly, the isolation campaigns revealed striking variations in recovering *S. cerevisiae* from grapes, as we isolated *S. cerevisiae* from <10% of the fermented grape bunches in CVPAO and LPAO areas. From PAO the yeast recovery was very high, as reflected by the percentage of *S. cerevisiae* on the total isolates. Despite their proximity, the occurrence of the three enological areas was strikingly different. The distribution of *S. cerevisiae* in CVPAO and LPAO sampled subareas was very irregular and with a low global occurrence (8.5 and 8.6% respective detection frequency). In contrast, PAO area, where Raboso red grape variety was cultivated, showed a homogenous distribution with frequent *S. cerevisiae* strains on grapes (69.2% of grapes samples). These findings showed a dramatically lower presence of *S. cerevisiae* in vineyard in CVPAO and LPAO than in other winemaking regions previously studied (Valero et al., 2007; Schuller et al., 2012).

The analysis of *S. cerevisae* intraspecific variability observed with mtDNA RFLP technique revealed that the lower the occurrence of S. *cerevisiae*, the lower the recovered mtDNA profile number. Therefore, we observed a low genotype number in LPAO and CVPAO with a ratio of 1:7 and 1:11 (number of mtDNA RFLP profile out of total *S. cerevisiae* isolates). In contrast to these two former areas, PAO was the winemaking region with the highest intraspecific variability with 129 different genotypes out of 295 total *S. cerevisiae* isolates (1:2). Schuller et al. (2012) evidenced an intermediate value of 1:5. In accordance with this author we found that a high percentage of fermenting samples per areas was associated with a high genotype number. As regards the number of different genotypes per spontaneous fermentation, most of the samples from CVPAO and LPAO showed a very limited number (with an average of 3 profiles per fermentation). In the case of PAO, although the variability was higher (up to 18 strains per fermented bunch with a average number of 9 different profiles), the genotype number was anyway lower than the one found by Schuller (frequently from 20 to 25, up to 41 in one case) or similar to that obtained by Valero (from 1 to 20) and Cordero-Bueso (from 1 to 21). The high diversity observed in PAO area (mean frequency of 8 genotypes) is comparable to that found in Portugal vineyards (Schuller et al., 2012). When we compared the mtDNA RFLP profiles with those of commercial strains, the profile percentage matching the industrial strains was rather surprising. The same industrial strains were analyzed through microsatellite typing and the results were compared with those of the vineyard isolates previously identified as industrial. Most of the vineyard isolates that shared the mtDNA profile with commercial strains confirmed a strong genetic relationship with the corresponding commercial strains, indicating a wide diffusion of commercial yeasts in the analyzed winemaking areas. Our results suggest that these isolates are derivatives of commercial strains that diffused from wineries to vineyards. The presence of industrial strains on grapes has been reported formerly (Valero et al., 2005, 2007; Martiniuk et al., 2016) but not at such high frequencies. Moreover, the dissemination of commercial yeasts was demonstrated to be restricted to short distances and limited periods of time (Valero et al., 2005, 2007). Our results suggested that commercial yeast dissemination can involve large winemaking areas where these yeasts spread permanently in the vineyard ecosystem. This was especially clear for CVPAO and indicates the real risk of loss of genetic diversity associated to the massive use of industrial starters (Cubillos et al., 2009).

In particular, the percentage of isolates that shared the same genetic profile of industrial strains reached 27.5% of the total and 51.5% in CVPAO. As regards the subarea distribution, only 4 subareas out of 11 in CVPAO and 4 out of 6 in LPAO, did not show the presence of industrial strains. Therefore, in CVPAO 63.6% of the subareas had a wide contamination of industrial strains that in Pr05 reached 100%. This is the first time that a so high level of industrial strains presence has been reported in such a big vineyard extension, highlighting industrial strain contamination as emerging problem in winemaking management. Similar contamination levels were previously found, but limited to a very small area (a single vineyard; Valero et al., 2005).

As regards PAO, the industrial genotype impact is comparable with those of other winemaking regions previously studied. Indeed, only 9.4% of total isolates in PAO had an industrial genotype. Our findings highlight that in industrial winemaking regions low commercial strain dissemination is associated to high autochthonous genotype diversity.

Among these industrial strains, the most frequent mtDNA profile was Lallemand Lalvin D47/Blastosel FR95, as it was found in all the sampled areas. In CVPAO 28.8% of *S. cerevisiae* isolates had this industrial profile that has been widely used in this area for primary fermentations for more than 10 years. This is in agreement with the clonal expansion of some strains and had been observed for strain D47, isolated in Tuscany in a previous work (Stefanini et al., 2012). We could also detect slight differences at few loci for some isolates, as for FR95. The presence of strains partially related to starters (i.e., P148.1 and FR95) is also in agreement with the proposal of the FAGE evolution model proposed by Sipiczki (2011), including clonal expansion, meiosis and recombination through intra-tetrade mating. Our results suggest that industrial strains diffusion significantly altered the spontaneous microbiota. This means that these strains are fit not only for the wine must fermentation environment, but also for the vineyard environment. Moreover, the presence of dominant industrial strains in different areas seem to be the result of clonal expansion of these strains, likely in human driven spontaneous fermentations. Microsatellite genotyping provided more information on yeast population structure. Indeed, when comparing the genotypes of the Italian vineyard isolates to strains isolated from other sources, such as laboratory, fermenting fruits, sake, or oak strains, we could notice isolate B169.12 that presented a high similarity with the oak isolate NC02 or isolate R149.1 with CLIB219 isolated from V*itis amurensis*. Hyma and Fay (2013) recently described such a case from an investigation of yeast diversity in USA vineyards. Three other isolates, R106.2, R106.3, and P254.2, were found similar to two beer yeasts: Clib382 and NCYC361. On the contrary, most of the other isolates co-clustered with industrial strains, or strains that had already been described as wine strains through their genome sequence (Liti et al., 2009; Novo et al., 2009; Borneman et al., 2011; Treu et al., 2014). In agreement with the wide use of industrial strain EC1118 and related strains QA23 and DV10, a cluster including 15 grape isolates and these yeast starters was observed.

Population inference enabled to distinguish several group of isolates. Two of the groups inferred for all runs, the first group contains EC1118 and isolates from the PAO area (i.e., R106.5) and other industrial strains; the second group contains the industrial strains MYC611 and FR95 and isolates from the three areas; the third and the largest group included 66 isolates from PAO and CVPAO areas, and two from LPAO, as well as the industrial strains L1414, VL3, LV10 and the wine strain L1374. Interestingly, the last group, which also presents the highest selfing rates, gathers 16 strains and includes oak strain NC02 and several isolates from LPAO area (i.e., T113b1, T314.1…) or PAO area (R102.1-3). Strikingly, it has been observed that natural isolates present a higher homozygosity than human-associated isolates (Diezmann and Dietrich, 2009), suggesting a different life style for the oak related isolates.

Regarding the geographical genotype distribution, the differences observed among the *S. cerevisiae* populations isolated from a wide sampling of the three Appellation of Origin area, are still puzzling. We observed an heterogeneous distribution of yeast populations with a clear PAO and LPAO transition. This might be associated to the different sampling year, but the presence of some sampling points from distant sites with different genotypes suggests that this is not solely a year/grape variety effect. The presence of transition zones is a reason pleading for a geographic differentiation: yeast population in Ra15 and also Ra17 and Ra11 (PAO) carry genotypes close to those of LPAO subareas. The presence of the isolates of the Cluster 12 (containing CLIB219-like strains) detected with InStruct in the three Appellation of Origin areas suggests also no clear-cut differences but more progressive variations in the different populations.

These results confirm the specificities of yeast microbiota at the vineyard scale, with a relative Appellation of Origin homogeneity, and transition zones. Geographical distance and grape varieties are apparently key elements involved in strain distribution, but the presence of industrial strains in the microbiota leads to the reduction of the genetic differentiation between populations. The differences in observed diversity between mtDNA-RFLP and microsatellite analysis may results from the smaller number of bands detected in the profiles (56) in comparison to 344 alleles detected at the 18 microsatellite loci. This suggests that mtDNA-RFLP underestimates the real genetic variance. Nevertheless, such differences may also result from the differences in inheritance between nuclear (biparental) and mitochondrial markers (uneven biparental), differences in the molecular mechanism of their changes (mutations in mtDNA and replication slippage for micro satellites) and differences in selection pressure.

In conclusion, this work indicates the geographical distance as one of the key elements, involved in differentiation of vineyard yeast populations. Surprisingly, we found that the dissemination of industrial yeasts in vineyard is a real emergency and their presence strongly interferes with the natural yeast microbiota. The industrial wine yeasts are considered human-domesticated strains due to fermentation process. In our work those found in vineyards are still so fit to the natural environment to take over indigenous populations. These results suggest that the human driven strain selection, during spontaneous fermentations, picked up almost randomly the clonal expanded genotype among those that better fit the vineyard environment.

## Author contributions

Principal investigator: AV. Designed research: VC, AG, AL. Samplings and fermentation trials: AV, MCa, CN. Strains identification and mtDNA RFLP analysis: AV, CN. Microsatellites amplification: AV, MCr, DM. Microsatellites and population structure analysis: JL. Wrote the manuscript: VC, JL. Edited the manuscript: VC, JL, AG, CN.

### Conflict of interest statement

The authors declare that the research was conducted in the absence of any commercial or financial relationships that could be construed as a potential conflict of interest. The reviewer RT and handling Editor declared their shared affiliation, and the handling Editor states that the process nevertheless met the standards of a fair and objective review.

## Supplementary material

Figure S1 Dendrogram obtained by mtDNA RFLP of the strains considered in the survey. The analysis was determined by the UPMGA method and performed by Dice similarity coefficient.

Figure S2 Neighbor net showing the clustering of 258 yeast strains isolated from different sources. The network was constructed from Bruvo's distance between strains based on the polymorphism at 18 loci. The names of the strains coming from our isolation programs are in black, and the 4 strains whose genome has been sequenced in pink. Branches of wine strains are in dark green for wine strains, except Champagne strains with EC1118 in light green, and the cluster of strains of LPAO area containing Clib219 in yellow/green. Names of commercial strains (in bold font) are in red for wine strains, and names of strains from various origins whose genome has been sequenced in light blue. Non wine strains are also indicated by purple branches.

Figure S3 Simultaneous plot of neighbor joining dendrograms obtained from each typing methods Microsatellite typing or mDNA RFLP after calculation of dissimilarity distance matrix between strains for each method.

Figure S4 Neighbour Joining tree presenting the differentiation of the different populations sampled in the three appellation areas measured by Fst (red PAO area, Brown LPAO area CVPAO area).

Table S1 Strains tested in this study.

Table S2 Weir and Cochran (1984) pairwise Fst between sampling areas and significance, calculated with Fstat. Pairwise Fst values are given in the lower triangular matrix and estimated *p*-values are given in the upper triangular matrix. Significance after correction of multiple tests at the 5% nominal level is indicated in bold (upper triangular matrix). NA: non-calcualted, NS: non-significant.

Table S3 Loadings of the different sampling site to the axis of SPCA.

Table S4 Microsatellite loci description and primers.

Table S5 Genotypes scored for each strain.
