# Supplementary figures and images for: The Geographic Distribution of Saccharomyces cerevisiae Isolates within three Italian Neighboring Winemaking Regions Reveals Strong Differences in Yeast Abundance, Genetic Diversity and Industrial Strain Dissemination
Source: Front Microbiol. 2017 Aug 24;8:1595. doi: 10.3389/fmicb.2017.01595 (PMC5573751; doi:10.3389/fmicb.2017.01595)

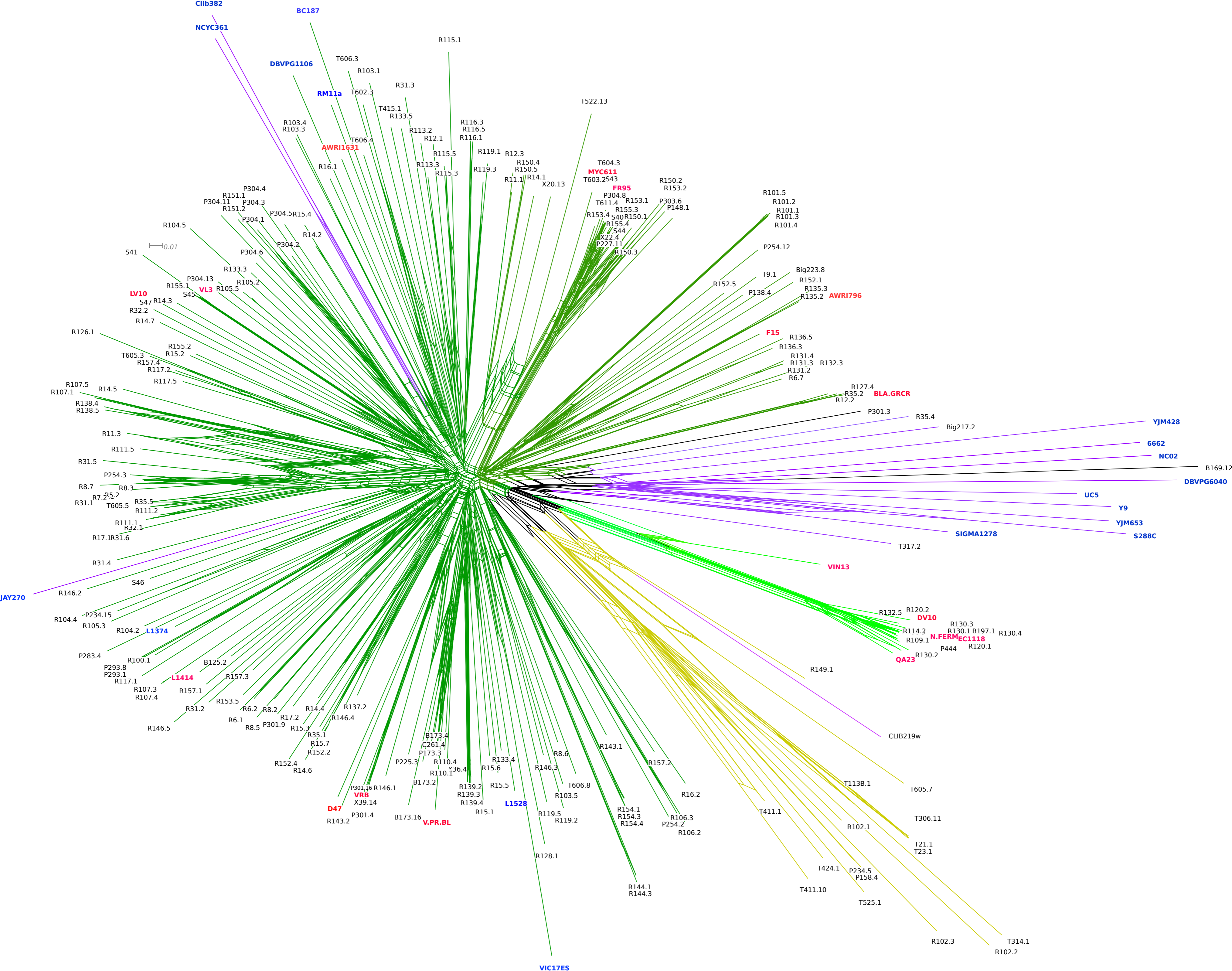

Supplement: Figure S2 — Neighbor net showing the clustering of 258 yeast strains isolated from different sources. The network was constructed from Bruvo's distance between strains based on the polymorphism at 18 loci. The names of the strains coming from our isolation programs are in black, and the 4 strains whose genome has been sequenced in pink. Branches of wine strains are in dark green for wine strains, except Champagne strains with EC1118 in light green, and the cluster of strains of LPAO area containing Clib219 in yellow/green. Names of commercial strains (in bold font) are in red for wine strains, and names of strains from various origins whose genome has been sequenced in light blue. Non wine strains are also indicated by purple branches. [file Image2.PDF]

Microsatellite

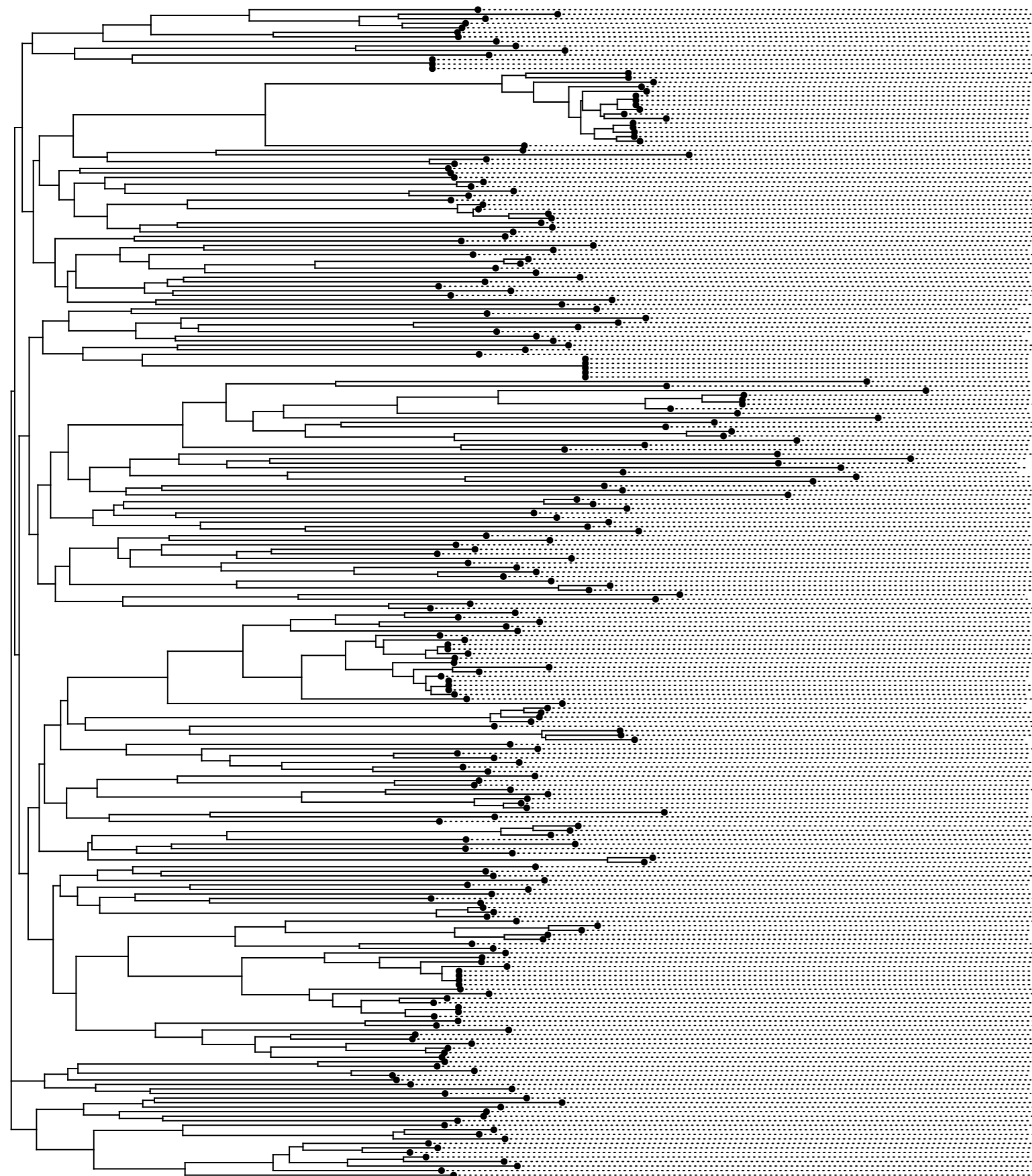

mtDNA RFLP

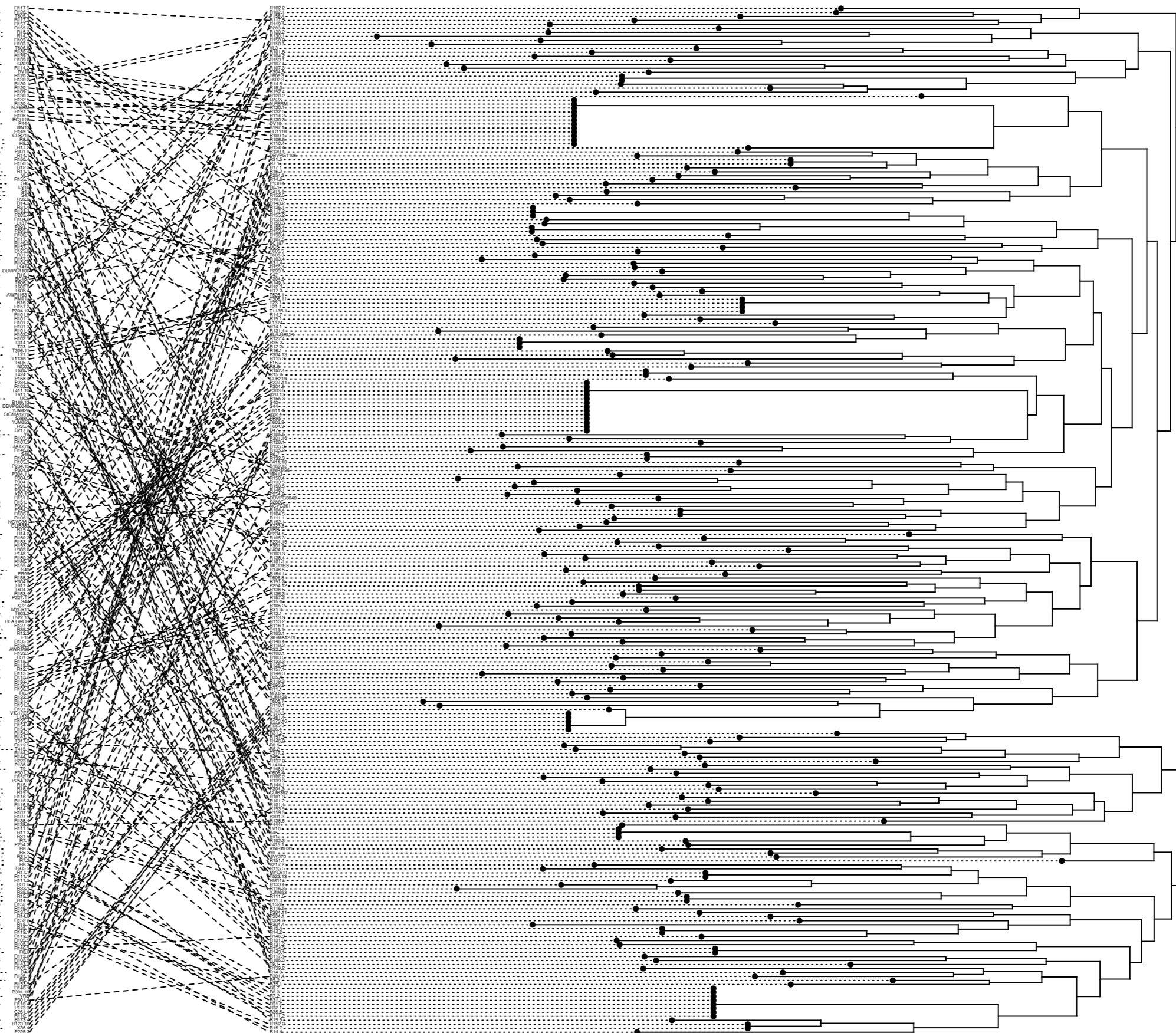

Supplement: Figure S3 — Simultaneous plot of neighbor joining dendrograms obtained from each typing methods Microsatellite typing or mDNA RFLP after calculation of dissimilarity distance matrix between strains for each method. [file Image3.PDF]

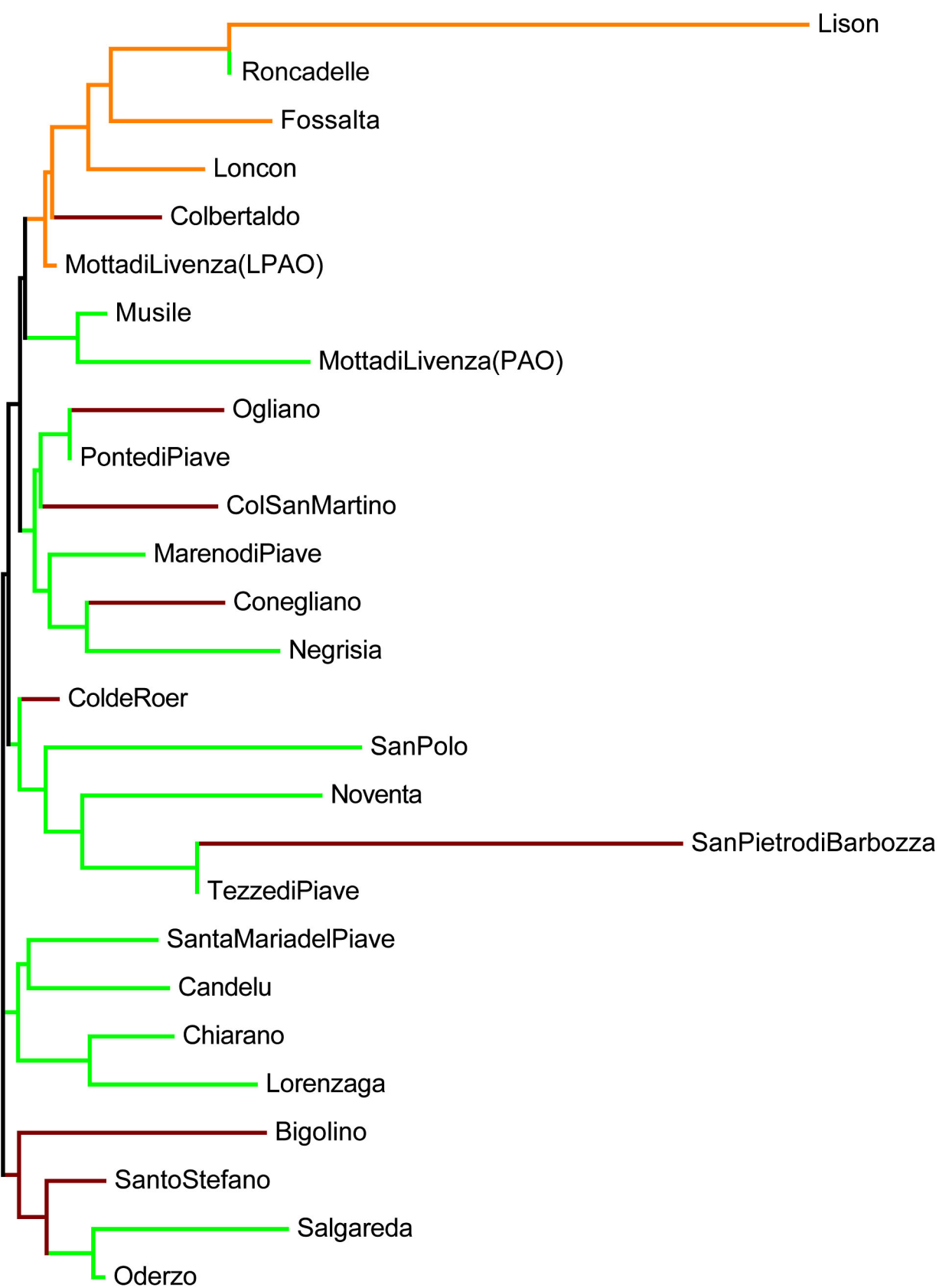

0.01

Supplement: Figure S4 — Neighbour Joining tree presenting the differentiation of the different populations sampled in the three appellation areas measured by Fst (red PAO area, Brown LPAO area CVPAO area). [file Image4.PDF]
