# Supplementary material for: The Geographic Distribution of Saccharomyces cerevisiae Isolates within three Italian Neighboring Winemaking Regions Reveals Strong Differences in Yeast Abundance, Genetic Diversity and Industrial Strain Dissemination
Source: Front Microbiol. 2017 Aug 24;8:1595. doi: 10.3389/fmicb.2017.01595 (PMC5573751; doi:10.3389/fmicb.2017.01595)
Supplement: Table S1 — Strains tested in this study. [file Table1.docx]

Table S1: Strains tested in this study

| CVPAO strain name | CVPAO subarea name | CVPAO subarea code |
| --- | --- | --- |
| B169.12 | Bigolino | Pr35 |
| B173.4 | Bigolino | Pr35 |
| P173.3 | Bigolino | Pr35 |
| B173.16 | Bigolino | Pr35 |
| B173.2 | Bigolino | Pr35 |
| P158.4 | Campea | Pr20 |
| P301.4 | Col de Roer | Pr34 |
| P301.9 | Col de Roer | Pr34 |
| P304.8 | Col de Roer | Pr34 |
| P303.6 | Col de Roer | Pr34 |
| P304.13 | Col de Roer | Pr34 |
| P304.6 | Col de Roer | Pr34 |
| P304.5 | Col de Roer | Pr34 |
| P304.3 | Col de Roer | Pr34 |
| P301.3 | Col de Roer | Pr34 |
| P304.2 | Col de Roer | Pr34 |
| P304.1 | Col de Roer | Pr34 |
| P304.4 | Col de Roer | Pr34 |
| P304.11 | Col de Roer | Pr34 |
| P148.1 | Col San Martino | Pr22 |
| B217.2 | Col San Martino | Pr22 |
| P138.4 | Col San Martino | Pr22 |
| P227.11 | Colbertaldo | Pr27 |
| P225.3 | Colbertaldo | Pr27 |
| P234.15 | Colbertaldo | Pr27 |
| P234.5 | Colbertaldo | Pr27 |
| S47 | Conegliano | Pr06 |
| S40 | Conegliano | Pr06 |
| S44 | Conegliano | Pr06 |
| S41 | Conegliano | Pr06 |
| S45 | Conegliano | Pr06 |
| S46 | Conegliano | Pr06 |
| X39.14 | Ogliano | Pr05 |
| X20.13 | Ogliano | Pr05 |
| X22b.4 | Ogliano | Pr05 |
| X36.4 | Ogliano | Pr05 |
| P293.1 | San Pietro di Barbozza | Pr31 |
| P293.8 | San Pietro di Barbozza | Pr31 |
| C261.1 | Santo Stefano | Pr24 |
| P283.4 | Santo Stefano | Pr24 |
| P254.3 | Santo Stefano | Pr24 |
| P254.2 | Santo Stefano | Pr24 |
| P254.12 | Santo Stefano | Pr24 |
| B197.1 | Santo Stefano | Pr24 |
| P219.1 | Scandolere | Pr26 |
| B223.8 | Scandolere | Pr26 |
| B125.2 | Susegana | Pr10 |
| B125.6 | Susegana | Pr10 |
| B125.5 | Susegana | Pr10 |
| B125.3 | Susegana | Pr10 |
| LPAO strains name | LPAO zone names | LPAO zone code |
| T411.1 | MottadiLivenza | To01 |
| T602.3 | Motta di Livenza | To01 |
| T606.3 | Motta di Livenza | To01 |
| T603.2 | Motta di Livenza | To01 |
| T604.3 | Motta di Livenza | To01 |
| T611.4 | Motta di Livenza | To01 |
| T605.3 | Motta di Livenza | To01 |
| T605.5 | Motta di Livenza | To01 |
| T605.7 | Motta di Livenza | To01 |
| T606.4 | Motta di Livenza | To01 |
| T606.8 | Motta di Livenza | To01 |
| T9.1 | Loncon | To05 |
| T113b.1 | Loncon | To05 |
| T317.2 | Loncon | To05 |
| T525.1 | Loncon | To05 |
| T21.1 | Lison | To08 |
| T23.1 | Lison | To08 |
| T306.11 | Lorenzaga | To02 |
| T411.10 | Annone Veneto | To06 |
| T415.1 | Fossalta | To13 |
| T424.1 | Fossalta | To13 |
| T522.13 | Fossalta | To13 |
| T314.1 | Fossalta | To13 |
| PAO strain name | PAO subarea name | PAO subarea code |
| R157.4 | Candelù | Ra05 |
| R157.1 | Candelù | Ra05 |
| R157.2 | Candelù | Ra05 |
| R157.3 | Candelù | Ra05 |
| 117.1 | Chiarano | Ra12 |
| R115.3 | Chiarano | Ra12 |
| R115.5 | Chiarano | Ra12 |
| R116.1 | Chiarano | Ra12 |
| R116.3 | Chiarano | Ra12 |
| R116.5 | Chiarano | Ra12 |
| R117.2 | Chiarano | Ra12 |
| R113.3 | Chiarano | Ra12 |
| R113.2 | Chiarano | Ra12 |
| R117.5 | Chiarano | Ra12 |
| R114.2 | Chiarano | Ra12 |
| R127.2 | Lorenzaga | Ra11 |
| R126.1 | Lorenzaga | Ra11 |
| R130.2 | Mansuè | Ra09 |
| R130.1 | Mansuè | Ra09 |
| R130.4 | Mansuè | Ra09 |
| R128.1 | Mansuè | Ra09 |
| R130.3 | Mansuè | Ra09 |
| R146.2 | Mareno di Piave | Ra01 |
| R146.4 | Mareno di Piave | Ra01 |
| R146.5 | Mareno di Piave | Ra01 |
| R146.1 | Mareno di Piave | Ra01 |
| R144.3 | Mareno di Piave | Ra01 |
| R144.1 | Mareno di Piave | Ra01 |
| R146.3 | Mareno di Piave | Ra01 |
| R119.5 | Motta di Livenza | Ra10 |
| R119.2 | Motta di Livenza | Ra10 |
| R119.1 | Motta di Livenza | Ra10 |
| R119.3 | Motta di Livenza | Ra10 |
| R120.2 | Motta di Livenza | Ra10 |
| R120.1 | Motta di Livenza | Ra10 |
| R104.5 | Musile | Ra16 |
| R104.2 | Musile | Ra16 |
| R105.2 | Musile | Ra16 |
| R105.5 | Musile | Ra16 |
| R104.4 | Musile | Ra16 |
| R105.1 | Musile | Ra16 |
| R103.1 | Musile | Ra16 |
| R103.3 | Musile | Ra16 |
| R103.5 | Musile | Ra16 |
| R103.4 | Musile | Ra16 |
| R155.4 | Negrisia | Ra06 |
| R154.3 | Negrisia | Ra06 |
| R154.1 | Negrisia | Ra06 |
| R154.4 | Negrisia | Ra06 |
| R155.2 | Negrisia | Ra06 |
| R155.3 | Negrisia | Ra06 |
| R155.1 | Negrisia | Ra06 |
| R101.1 | Noventa | Ra15 |
| R100.1 | Noventa | Ra15 |
| R101.3 | Noventa | Ra15 |
| R101.4 | Noventa | Ra15 |
| R101.2 | Noventa | Ra15 |
| R101.5 | Noventa | Ra15 |
| R102.2 | Noventa | Ra15 |
| R102.1 | Noventa | Ra15 |
| R102.3 | Noventa | Ra15 |
| R31.5 | Oderzo | Ra08 |
| R31.1 | Oderzo | Ra08 |
| R35.2 | Oderzo | Ra08 |
| R31.6 | Oderzo | Ra08 |
| R32.1 | Oderzo | Ra08 |
| R35.5 | Oderzo | Ra08 |
| R32.2 | Oderzo | Ra08 |
| R35.1 | Oderzo | Ra08 |
| R31.4 | Oderzo | Ra08 |
| R31.3 | Oderzo | Ra08 |
| R35.4 | Oderzo | Ra08 |
| R31.2 | Oderzo | Ra08 |
| R152.2 | Ponte di Piave | Ra07 |
| R153.4 | Ponte di Piave | Ra07 |
| R150.2 | Ponte di Piave | Ra07 |
| R153.2 | Ponte di Piave | Ra07 |
| R150.3 | Ponte di Piave | Ra07 |
| R153.1 | Ponte di Piave | Ra07 |
| R150.1 | Ponte di Piave | Ra07 |
| R151.2 | Ponte di Piave | Ra07 |
| R150.4 | Ponte di Piave | Ra07 |
| R150.5 | Ponte di Piave | Ra07 |
| R152.1 | Ponte di Piave | Ra07 |
| R152.4 | Ponte di Piave | Ra07 |
| R152.5 | Ponte di Piave | Ra07 |
| R153.5 | Ponte di Piave | Ra07 |
| R149.1 | Ponte di Piave | Ra07 |
| R151.1 | Ponte di Piave | Ra07 |
| R12.2 | Roncadelle | Ra04 |
| R11.1 | Roncadelle | Ra04 |
| R12.1 | Roncadelle | Ra04 |
| R12.3 | Roncadelle | Ra04 |
| R14.4 | Roncadelle | Ra04 |
| R14.5 | Roncadelle | Ra04 |
| R14.7 | Roncadelle | Ra04 |
| R15.1 | Roncadelle | Ra04 |
| R15.2 | Roncadelle | Ra04 |
| R15.5 | Roncadelle | Ra04 |
| R16.1 | Roncadelle | Ra04 |
| R16.2 | Roncadelle | Ra04 |
| R17.2 | Roncadelle | Ra04 |
| R15.7 | Roncadelle | Ra04 |
| R14.1 | Roncadelle | Ra04 |
| R14.2 | Roncadelle | Ra04 |
| R14.3 | Roncadelle | Ra04 |
| R17.1 | Roncadelle | Ra04 |
| R11.3 | Roncadelle | Ra04 |
| R15.3 | Roncadelle | Ra04 |
| R14.6 | Roncadelle | Ra04 |
| R15.6 | Roncadelle | Ra04 |
| R15.4 | Roncadelle | Ra04 |
| R111.2 | Salgareda | Ra14 |
| R111.5 | Salgareda | Ra14 |
| R111.1 | Salgareda | Ra14 |
| R110.1 | Salgareda | Ra14 |
| R110.4 | Salgareda | Ra14 |
| R109.1 | Salgareda | Ra14 |
| R107.3 | San Polo | Ra03 |
| R107.4 | San Polo | Ra03 |
| R107.5 | San Polo | Ra03 |
| R106.3 | San Polo | Ra03 |
| R106.2 | San Polo | Ra03 |
| R107.1 | San Polo | Ra03 |
| R106.5 | San Polo | Ra03 |
| R139.2 | Santa Maria del Piave | Ra17 |
| R139.3 | Santa Maria del Piave | Ra17 |
| R139.4 | Santa Maria del Piave | Ra17 |
| R137.2 | Santa Maria del Piave | Ra17 |
| R143.2 | Santa Maria del Piave | Ra17 |
| R143.1 | Santa Maria del Piave | Ra17 |
| R138.5 | Santa Maria del Piave | Ra17 |
| R138.4 | Santa Maria del Piave | Ra17 |
| R8.3 | Tezze di Piave | Ra02 |
| R7.2 | Tezze di Piave | Ra02 |
| R6.1 | Tezze di Piave | Ra02 |
| R5.2 | Tezze di Piave | Ra02 |
| R8.2 | Tezze di Piave | Ra02 |
| R7.1 | Tezze di Piave | Ra02 |
| R8.7 | Tezze di Piave | Ra02 |
| R8.6 | Tezze di Piave | Ra02 |
| R6.7 | Tezze di Piave | Ra02 |
| R132.3 | Tezze di Piave | Ra02 |
| R133.4 | Tezze di Piave | Ra02 |
| R133.5 | Tezze di Piave | Ra02 |
| R136.3 | Tezze di Piave | Ra02 |
| R136.5 | Tezze di Piave | Ra02 |
| R131.4 | Tezze di Piave | Ra02 |
| R131.3 | Tezze di Piave | Ra02 |
| R131.2 | Tezze di Piave | Ra02 |
| R135.3 | Tezze di Piave | Ra02 |
| R135.2 | Tezze di Piave | Ra02 |
| R133.3 | Tezze di Piave | Ra02 |
| R132.5 | Tezze di Piave | Ra02 |
| NAME | ORIGIN | CODE |
| AWRI1631 | Wine, AUSTRALIA (sequenced) | wine |
| AWRI796 | Wine, AUSTRALIA (sequenced) | wine |
| BC187 | Wine, USA (sequenced) | wine |
| BLA.GRCR | Wine, EUROPE | wine |
| CLIB219w | *Vitis amurensis*, East Russia | Oak/nature |
| D47 | Wine, EUROPE | wine |
| DV10 | Wine, EUROPE | wine |
| EC1118 | Wine, EUROPE (sequenced) | wine |
| F15 | Wine, EUROPE | wine |
| FR95 | Wine, EUROPE | wine |
| JAY270 | Sugar Cane fermentation, Brazil | Rum |
| L1374 | Wine, Chile (sequenced) | wine |
| L1414 | Wine, Europe | wine |
| L1528 | Wine, Chile (sequenced) | wine |
| LV10 | Wine, EUROPE | wine |
| MYC611 | Wine, EUROPE | wine |
| N.FERM | Wine, EUROPE | wine |
| P444 | Wine, EUROPE | wine |
| QA23 | Wine, EUROPE (sequenced) | wine |
| RM11.1a | Wine, EUROPE (sequenced) | wine |
| V.PR.BL | Wine, EUROPE | wine |
| IIC17ES | Wine, EUROPE (sequenced) | wine |
| VIN13 | Wine, EUROPE (sequenced) | wine |
| VL3 | Wine, EUROPE (sequenced) | wine |
| VRB | Wine, EUROPE | wine |
| DBVPG1106 | Fermented fruit, EUROPE (sequenced) | fruit |
| DBVPG6040 | Fermented fruit, EUROPE (sequenced) | fruit |
| Y9 | Ragi, ASIA | ragi |
| UC5 | Sake, ASIA | sake |
| Clib382 | Beer, EUROPE | beer |
| NCYC361 | Beer, EUROPE | beer |
| 6662 | Bread, EUROPE | bread |
| YJM428 | Clinical isolate, USA | Is.Cl |
| YJM653 | Clinical isolate, USA | Is.Cl |
| S288C | Laboratory, USA | lab |
| SIGMA1278 | Laboratory (sequenced) | lab |
| NC02 | oak tree exudates, North Oak, Caroline, USA | oak |

Table S2 (*see excel file*): Weir and Cochran (1984) pairwise Fst between sampling areas and significance, calculated with Fstat. Pairwise Fst values are given in the lower triangular matrix and estimated pvalues are given in the upper triangular matrix. Significance after correction of multiple tests is indicated by bold figures in the upper triangular matrix. NA: Non-calculated, NS: not significant. Significances are indicated after correction for multiple testing.
