# Supplementary material for: The Geographic Distribution of Saccharomyces cerevisiae Isolates within three Italian Neighboring Winemaking Regions Reveals Strong Differences in Yeast Abundance, Genetic Diversity and Industrial Strain Dissemination
Source: Front Microbiol. 2017 Aug 24;8:1595. doi: 10.3389/fmicb.2017.01595 (PMC5573751; doi:10.3389/fmicb.2017.01595)
Supplement: Table S3 — Loadings of the different sampling site to the axis of SPCA. [file Table3.DOCX]

Table S3: Loadings of the different sampling site to the axis of SPCA.

| Appellation |  | Axis 1 | Axis 2 | Axis 3 | Axis 26 |
| --- | --- | --- | --- | --- | --- |
| LPAO | To05 | 0.84 | 0.11 | -0.29 | -0.08 |
|  | To08 | 0.69 | 0.04 | 0.01 | 0.11 |
|  | To13 | 0.81 | -0.07 | 0.28 | -0.10 |
|  | To01 | -0.05 | 0.30 | 0.10 | 0.05 |
| CVPAO | Pr35 | -0.13 | -0.54 | -0.12 | 0.49 |
|  | Pr24 | -0.18 | -0.03 | 0.36 | -0.24 |
|  | Pr22 | -0.25 | -0.44 | 0.37 | 0.39 |
|  | Pr27 | -0.36 | -0.42 | -0.06 | 0.22 |
|  | Pr31 | -0.14 | -0.45 | 0.01 | -0.56 |
|  | Pr34 | -0.41 | -1.21 | -0.31 | 0.73 |
|  | Pr06 | -0.30 | 0.13 | 0.48 | -0.19 |
|  | Pr05 | -0.02 | 0.14 | 0.34 | 0.07 |
| PAO | Ra15 | -0.25 | 0.13 | -0.19 | -0.02 |
|  | Ra16 | 0.59 | -0.15 | -0.18 | -0.22 |
|  | Ra03 | -0.39 | 0.27 | -0.17 | 0.12 |
|  | Ra04 | -0.47 | 0.23 | -0.18 | 0.15 |
|  | Ra14 | -0.25 | 0.30 | -0.32 | 0.07 |
|  | Ra12 | 0.16 | 0.18 | -0.18 | 0.09 |
|  | Ra10 | 0.73 | 0.13 | 0.11 | -0.25 |
|  | Ra11 | 1.07 | 0.11 | -0.11 | -0.04 |
|  | Ra02 | -0.36 | 0.08 | -0.19 | 0.00 |
|  | Ra17 | -0.26 | 0.04 | 0.26 | 0.07 |
|  | Ra01 | -0.38 | 0.24 | 0.12 | -0.06 |
|  | Ra07 | -0.40 | 0.21 | -0.22 | 0.09 |
|  | Ra06 | -0.37 | 0.21 | -0.17 | 0.07 |
|  | Ra05 | -0.22 | 0.01 | -0.13 | -0.16 |
|  | Ra08 | -0.31 | 0.26 | 0.04 | -0.14 |
