# Supplementary material for: The Geographic Distribution of Saccharomyces cerevisiae Isolates within three Italian Neighboring Winemaking Regions Reveals Strong Differences in Yeast Abundance, Genetic Diversity and Industrial Strain Dissemination
Source: Front Microbiol. 2017 Aug 24;8:1595. doi: 10.3389/fmicb.2017.01595 (PMC5573751; doi:10.3389/fmicb.2017.01595)
Supplement: Table S4 — Microsatellite loci description and primers. [file Table4.docx]

Table S4: Microsatellite loci description and primers

| **Locus name** | **Motif** | **ORF or coordinates** | **Primers** | **Dye** |
| --- | --- | --- | --- | --- |
| MIX 1 |  |  |  |  |
| C3 | CAA | YGL139w | FW-CTTTTTATTTACGAGCGGGCCAT  RV-AAATCTCATGCCTGTGAGGGGTAT | NED |
| C5 | GT | VI-210250/  210414 | FW-TGACACAATAGCAATGGCCTTCA  RV-GCAAGCGACTAGAACAACAATCACA | VIC |
| C8 | TAA | YGL014w | FW-CAGGTCGTTCTAACGTTGGTAAAATG  RV- GCTGTTGCTGTTGGTAGCATTACTGT | FAM |
| C11 | GT | X-518870/  519072 | FW-TTCCATCATAACCGTCTGGGATT  RV-TGCCTTTTTCTTAGATGGGCTTTC | FAM |
| YKR072c | GAC | YKR072c | FW-AGATACAGAAGATAAGAACGAAAA  RV-TTATTGATGCTTATCTATTATACC | PET |
| SCYOR267c | TGT | YOR267c | FW-TACTAACGTCAACACTGCTGCCAA  RV-GGATCTACTTGCAGTATACGGG | VIC |
| SCAAT2 | TAA | YBL084c | FW-CAGTCTTATTGCCTTGAACGA  RV-GTCTCCATCCTCCAAACAGCC | PET |
| SCAAT3 | TAA | YDR160w | FW-TGGGAGGAGGGAAATGGACAG  RV-TTCAGTTACCCGCACAATCTA | NED |
| SCAAT6 | TAA | IX-105711/  105883 | FW-TTACCCCTCTGAATGAAAACG  RV-AGGTAGTTTAGGAAGTGAGGC | PET |
| MIX 2 |  |  |  |  |
| SCAAT5 | TAA | XVI-897051/  8970210 | FW-AGCATAATTGGAGGCAGTAAAGCA  RV-TCTCCGTCTTTTTTGTACTGCGTG | NED |
| C4 | TAA  +TAG | (i) XV-110701/110935 | FW-AGGAGAAAAATGCTGTTTATTCTGACC  RV- TTTTCCTCCGGGACGTGAAATA | NED |
| C6 | CA | XVI-485898/  485996 | FW-GTGGCATCATATCTGTCAATTTTATCAC  RV-VIC-CAATCAAGCAAAAGATCGGCCT | VIC |
| YPL009c | CTT | YPL009c | FW-AACCCATTGACCTCGTTACTATCGT  RV-TTCGATGGCTCTGATAACTCCATTC | FAM |
| C9 | TAA | YOR156c | FW-AAGGGTTCGTAAACATATAACTGGCA  RV-TATAAGGGAAAAGAGCACGATGGC | NED |
| SCAAT1 | TTA | XIII-86902/  87140 | FW-AAAGCGTAAGCAATGGTGTAGATACTT  RV-CAAGCCTCTTCAAGCATGACCTTT | VIC |
| YKL172w | GAA | YKL172w | FW-CAGGACGCTACCGAAGCTCAAAAG  RV-ACTTTTGGCCAATTTCTCAAGAT | FAM |
| YLR | TC | XII-823393/  823562 | FW-CTGGAATGAAATTAAACAAAAGC  RV-TCTTCCTTTTCTACTATCTTCTC | PET |
| YLL049 | TA | XII | FW-GCAACATAATGATTTTGAGGT  RV-GTGTCTTGTGTGAGCATAGTGGAGAA | PET |

Table S5: Amplicon sizes of the 18 loci analysed (*see excel file*)
